# L’Envoi

**Published:** 1914-05

**Authors:** Rudyard Kipling


### L'Envoi.

When Earth's last picture is painted
And the tubes are twisted and dried,
When the oldest colors have faded,
And the youngest critic has died,
We shall rest—and, faith, we shall need it—
Lie down for an eon or two,
Till the Master of All good Workmen
Shall set us to work anew!
And only the Master shall praise us
And only the Master shall blame;
And no one shall work for money,
And no one shall work for fame;
But each for the joy of the working
And each in his separate star
Shall draw the Thing as he sees it
For the God of Things as They are.

—*Rudyard Kipling.*

